# Clinical‐Grade Human Induced Pluripotent Stem Cell‐Derived Neural Precursor Cells Restore Motor Function and Preserve Striatal Integrity in a Quinolinic Acid‐Lesioned Rat Model of Huntington's Disease

**DOI:** 10.1111/cpr.70189

**Published:** 2026-02-26

**Authors:** Hyeonjoong Jeon, Il‐Shin Lee, Sanghun Lee, Hyo Chang Park, Beomsoo Kim, Hyun Sook Kim, Jihwan Song

**Affiliations:** ^1^ Department of Biomedical Sciences CHA University Seongnam‐si Gyeonggi‐do Republic of Korea; ^2^ iPS Bio, Inc. Seongnam‐si Gyeonggi‐do Republic of Korea; ^3^ Department of Neurology, CHA Bundang Medical Center CHA University Seongnam‐si Gyeonggi‐do Republic of Korea

**Keywords:** HLA‐homozygous, Huntington's disease, induced pluripotent stem cells, medium spiny neurons, neural precursor cells, neuroinflammation, quinolinic acid (QA)

## Abstract

Huntington's disease (HD) is an inherited neurodegenerative disease characterised by progressive degeneration of GABAergic medium spiny neurons (MSNs) in the striatum. Neural precursor cells (NPCs) derived from human induced pluripotent stem cells (iPSCs) have been considered as a promising and scalable source for neuronal replacement and circuit restoration. In this study, we investigated the therapeutic effects of a clinical‐grade, human leukocyte antigen (HLA)‐homozygous iPSC line (YZWJ‐s513) differentiated into NPCs (s513‐NPCs) in a quinolinic acid (QA)‐lesioned rat model of HD. Following intrastriatal transplantation, s513‐NPCs not only survived for 12 weeks but also differentiated into neurons, astrocytes, and oligodendrocytes, while generating new DARPP32^+^ GABAergic MSNs. Specifically, graft‐derived neurons projected to the host globus pallidus, indicating structural integration into the striato‐pallidal pathways. Additionally, NPC‐transplanted rats exhibited significant motor recovery across multiple tasks for up to 12 weeks, accompanied by reduced striatal atrophy and ventricular enlargement. Histological findings also revealed attenuated astrogliosis and microgliosis, along with a shift toward an anti‐inflammatory milieu. Collectively, these results demonstrate that transplantation of clinical‐grade, HLA‐homozygous iPSC‐derived NPCs can provide both neuronal replacement and modulation of the diseased microenvironment, supporting their potential as a regenerative therapy for HD. Key quality attributes and release criteria supporting the clinical‐grade characterisation of the cell product used in vivo are summarised in Table S1.

## Graphical abstract

Clinical‐grade HLA‐homozygous iPSC‐derived neural precursor cells restore motor function, rebuild striatal circuitry and reduce neuroinflammation in QA‐lesioned rats. These findings demonstrate robust neuronal replacement and microenvironment modulation, supporting their potential as a regenerative therapy for Huntington's disease.

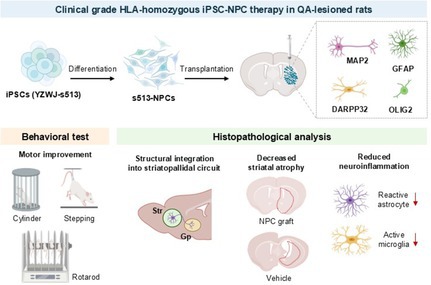

## 1 Introduction

Huntington's disease (HD) is an autosomal dominant neurodegenerative disease that manifests as progressive impairment in motor control, cognitive faculties, and psychiatric stability [1, 2, 3, 4, 5]. It is caused by an expanded CAG trinucleotide repeat in the *huntingtin* (*HTT*) gene, which leads to the production of mutant huntingtin protein (mHTT) and the selective degeneration of GABAergic medium spiny neurons (MSNs) in the striatum [1, 5, 6, 7, 8, 9]. Despite extensive efforts, no effective disease‐modifying treatment has been approved [10]. Recent trials targeting mHTT lowering, such as antisense oligonucleotides and RNA interference, have failed to show consistent therapeutic benefit [10, 11, 12]. Consequently, regenerative medicine approaches, including human pluripotent stem cell‐based therapy, have gained increasing attention as alternative strategies for HD [7, 13].

iPSCs provide a renewable and ethically feasible cell source for therapeutic application [13, 14, 15]. However, autologous iPSC therapy is hindered by production cost, time, and individualised manufacturing [13, 14]. As an alternative, HLA‐homozygous iPSC lines have been proposed to allow for immune‐compatible allogeneic transplantation with reduced immunosuppressive burden [16, 17, 18, 19]. In this study, we employed YZWJ‐s513, a clinical‐grade, HLA‐homozygous iPSC line derived from a donor representing a common HLA haplotype in East Asian populations, to generate NPCs for preclinical evaluation in a quinolinic acid (QA)‐lesioned rat model of HD. To strengthen translational interpretation, we also consider striatal structural preservation and inflammatory modulation as clinically meaningful intermediate phenotypes that align with broader brain‐structure‐to‐behaviour frameworks described in large‐cohort neuroimaging and psychiatric biomarker studies (conceptually complementary rather than disease‐specific). Beyond disease‐specific pathology, increasing evidence from neurodevelopmental and psychiatric research highlights how local neural microenvironments and structural integrity shape behavioural phenotypes, providing a broader conceptual framework for linking graft‐mediated microenvironmental modulation to functional outcomes.

QA, an excitotoxin and NMDA receptor agonist, induces MSN‐specific striatal degeneration while sparing afferent projections, thereby mimicking essential pathological and behavioural features of HD [20, 21, 22, 23, 24]. Using this model, we investigated whether transplantation of s513‐derived NPCs could replace lost neurons, restore striatal circuitry, and exert neuroprotective and immunomodulatory effects. Our results demonstrate that s513‐NPCs engraft, differentiate into GABAergic MSNs and glial lineages, structurally integrate into host circuitry, and significantly improve motor behaviour and neuropathology in an HD rodent model.

## 2 Materials and Methods

### 2.1 Clinical‐Grade iPSC Line

The clinical‐grade human iPSC line, YZWJ‐s513, was used in all experiments [25]. This line was derived from umbilical cord blood of an HLA‐homozygous donor (HLA‐A*24:02, B*52:01, DRB1*15:02) and established under Good Manufacturing Practice (GMP)‐compliant conditions by the CiRA Foundation (Kyoto, Japan). The clinical‐grade NPC product used for in vivo transplantation met predefined release criteria, including cell viability, identity, purity, residual pluripotency, genetic stability, sterility, mycoplasma, and endotoxin levels. Quantitative acceptance thresholds and testing methods for each release parameter are summarised in Table S1. All release testing was performed according to GMP‐compliant standard operating procedures for the specific batch used in vivo (see Table S1 for thresholds).

Although formal batch‐to‐batch variability testing was not conducted within the scope of this study, NPCs were manufactured from a single clinical‐grade master cell bank using standardised SOPs, supporting scalability and manufacturing reproducibility. In addition, while no single in vitro assay has been formally qualified as a definitive potency test for this NPC product, reproducible neuronal differentiation capacity (including MAP2^+^ and DARPP32^+^ lineage commitment) and consistent in vivo survival and behavioural efficacy were considered functional surrogates of potency at the preclinical stage.

### 2.2 iPSC Culture and Expansion

YZWJ‐s513 iPSCs were maintained under feeder‐free conditions on iMatrix‐511 (Matrixome, Japan)‐coated plates in StemFit Basic 03 medium (Ajinomoto, Japan) supplemented with 80 ng/mL recombinant human basic fibroblast growth factor (bFGF). Cultures were incubated at 37°C with 5% CO_2_ and passaged every 5 days using TrypLE Select (Gibco). To enhance survival after dissociation, 10 μM Y‐27632 (ROCK inhibitor, PeproTech) was included for the first 24 h post‐seeding.

### 2.3 Neural Induction and Derivation of NPCs

NPCs were generated using a modified embryoid body (EB)‐based differentiation protocol optimised for striatal lineage specification [26, 27]. Single‐cell suspensions of iPSCs were seeded at 6000 cells/microwell in AggreWell 800 plates (STEMCELL Technologies) with StemFit Basic 03 medium containing 10 μM Y‐27632 and 80 ng/mL bFGF. After 24 h, the medium was replaced with neural induction medium (NIM; DMEM/F12, 5% KnockOut Serum Replacement, 0.1 mM non‐essential amino acids, 0.05 mM β‐mercaptoethanol, 100 nM LDN‐193189, and 10 μM SB431542). NIM was refreshed every other day for 8 days. On day 9, EBs were dissociated with Accutase and plated on iMatrix‐511 in Neural Maintenance Medium (NMM; 1:1 DMEM/F12 and neurobasal medium, N2, B27 without vitamin A and 40 ng/mL bFGF). NPCs were expanded by passaging every 5 days.

### 2.4 Flow Cytometry Analysis

Cells were dissociated with 0.5 mM EDTA and stained with PE‐conjugated antibodies against pluripotency markers (SSEA4, TRA‐1‐60, TRA‐1‐81), NPC markers (PSA‐NCAM, NESTIN), and neural crest markers (HNK‐1, CD271) (Miltenyi Biotec). Isotype controls (IgG1, IgM) were used for specificity. Data were acquired using a BD Accuri C6 Plus Flow Cytometer and analysed with BD Accuri software (v1.0.34.1).

### 2.5 QA‐Lesioned Rat Model of HD and Cell Transplantation

All animal procedures were approved by the Institutional Animal Care and Use Committee of CHA University (IACUC 220047). Rats were housed 2–3 per cage in a temperature‐ and humidity‐controlled room (22°C ± 2°C; 12 h light/dark cycle) with free access to food and water. Eight‐week‐old male Sprague–Dawley rats (JA Bio, Korea) received unilateral intrastriatal injections of QA (120 nmol in 2 μL PBS, Sigma) at the following stereotaxic coordinates relative to bregma: AP +1.0 mm, ML +2.5 mm, DV –5.0 mm, as described previously [28]. One week later, 2 × 10^5^ YZWJ‐s513‐derived NPCs in 2 μL HBSS were transplanted into the ipsilateral striatum at AP +1.5 mm, ML +3.0 mm, DV –5.0 mm, while control animals received HBSS only. Rats were randomly assigned to NPC‐transplanted (*n* = 10) or vehicle (*n* = 10) groups. The sample size (*n* = 10 per group) was chosen based on previous QA‐lesioned rat transplantation studies using similar group sizes, which were sufficient to detect treatment‐related differences in motor behaviour and histological outcomes. All animals that underwent QA lesion and transplantation surgery and survived to the planned endpoint were included in the study; no animals or data points were excluded based on pre‐established criteria. Cyclosporine A (CsA) (Chong Kun Dang, Korea) was administered intraperitoneally at 10 mg/kg daily starting 2 days before transplantation and maintained at 5 mg/kg/day thereafter. All in vivo experiments were designed and reported in accordance with the ARRIVE 2.0 guidelines for animal research. Animals were assigned using a computer‐generated random number sequence (block randomisation, block size = 4), generated by an investigator not involved in surgeries or outcome assessments. Behavioural testing was performed by investigators blinded to group allocation. Because stereotaxic transplantation necessarily involves awareness of treatment allocation, experimenters were not blinded during the injection procedure; however, all downstream behavioural testing and histological quantification were performed by investigators blinded to group allocation. All histological quantification was performed by observers blinded to group allocation.

### 2.6 Adeno‐Associated Virus (AAV)‐Mediated Retrograde Tracing

To assess graft‐to‐host connectivity, a retrograde AAV vector encoding mCherry under the CAG promoter (AAVrg‐CAG‐mCherry, SignaGen Laboratories, Cat# SL116052) was injected into the globus pallidus (2 μL, 2.5 × 10^9^ viral genomes/mL) at 10 weeks post‐transplantation (coordinates: AP +1.5 mm, ML +3.5 mm, DV –6.0 mm) [29]. Animals were sacrificed 2 weeks later for histological analysis.

### 2.7 Behavioural Assessments

Motor function was assessed biweekly over 12 weeks by blinded investigators using an accelerating rotarod (5–40 rpm over 120 s, Panlab, Spain) to measure latency to fall as the average of three trials [30], a stepping test on a motorised treadmill (23 rpm) to count adjusting steps over 10 s [31] and a cylinder test to quantify forelimb use asymmetry by recording wall contacts during spontaneous vertical exploration in a transparent cylinder [30].

### 2.8 Immunocytochemistry and Immunohistochemistry

For in vitro staining, cells were fixed with 4% paraformaldehyde (PFA) for 15 min. For in vivo experiments, rats were perfused with PBS followed by 4% PFA at 12 weeks post‐transplantation. Brains were post‐fixed, cryoprotected in 30% sucrose, embedded in OCT, and sectioned coronally at 40 μm. Sections were blocked in PBS with 5% normal donkey serum and 0.3% Triton X‐100, incubated overnight with primary antibodies, and visualised with Alexa Fluor‐conjugated secondary antibodies. Nuclei were counterstained with DAPI. Imaging was performed on a Zeiss LSM880 confocal or Nikon Eclipse Ni fluorescence microscope. Image analysis was performed in ImageJ. Primary antibodies used included: goat anti‐Nanog (R&D system, 1:500), mouse anti‐OCT4 (Santa Cruz, 1:500), rabbit anti‐SOX2 (Millipore, 1:500), mouse anti‐NESTIN (R&D system, 1:500), mouse anti‐hNuclei (Millipore, 1:500), rabbit anti‐MAP2 (Abcam, 1:500), rabbit anti‐GFAP (Dako, 1:500), mouse anti‐OLIG2 (Sigma, 1:500), rabbit anti‐DARPP32 (Abcam, 1:500), rabbit anti‐hDARPP32 (Abcam, 1:500), mouse anti‐GAD65/67 (Santa Cruz, 1:500), goat anti‐KU80 (R&D Systems, 1:500), rabbit anti‐IBA1 (WAKO, 1:500), rabbit antiNOS (Thermo Fisher Scientific, 1:500), mouse anti‐ED1 (Abcam, 1:500) and mouse anti‐CD206 (Santa Cruz, 1:500).

### 2.9 Histological Quantification

Quantitative analysis of graft survival, cellular differentiation, and host tissue responses was performed on serial coronal brain sections from transplanted rats [32, 33]. Five 40 μm sections per animal, spanning +2.5 to −1.9 mm AP relative to bregma, were analysed. Graft survival was assessed by counting the number of hNuclei^+^/KU80^+^ double‐positive cells within the graft core at ×40 magnification. Neuronal differentiation was evaluated by calculating the percentage of cells co‐expressing human nuclear markers and neuronal lineage markers such as DARPP32, MAP2, and GAD67. Striatal atrophy was quantified by measuring the cross‐sectional area of the ipsilateral versus contralateral striatum under ×10 magnification. Host immune responses were evaluated by determining the proportion of iNOS‐positive or CD206‐positive cells among total IBA1‐ or ED1‐positive microglia/macrophages in five predefined regions of interest within the striatal graft boundary, as illustrated in Figure 6A. Serial sections spanning the graft were screened for rosette‐like structures, cysts, and proliferative foci; no gross overgrowth or teratoma‐like structures were detected within the 12‐week observation period. For endpoint histological analyses, brain tissue was available from all vehicle‐treated rats (*n* = 10) and from a subset of NPC‐transplanted rats (*n* = 6), whereas all 10 animals per group were included in the behavioural assessments. In four NPC‐transplanted animals, tissue integrity at these predefined levels was insufficient for quantitative histology, resulting in a reduced histological sample size while all animals remained eligible for longitudinal behavioural analyses. All analyses were performed by blinded observers using ImageJ. Importantly, behavioural outcomes were assessed longitudinally in the full cohort prior to tissue processing, reducing the likelihood that histological attrition biased functional conclusions.

### 2.10 Statistical Analysis

Data are presented as means ± standard error of the mean (SEM). Two‐group comparisons for endpoint histological measures were performed using unpaired *t*tests. Time‐course behavioural data were analysed using two‐way repeated‐measures ANOVA with factors of treatment and time, followed by Bonferroni post hoc tests for multiple comparisons. An individual animal was considered the experimental unit for all analyses. Statistical analyses were performed in GraphPad Prism version 5.01 (GraphPad Software, San Diego, CA), and significance was set at *p* < 0.05. Assumptions of normality and sphericity were examined and not violated. Normality was assessed using the Shapiro–Wilk test, and sphericity was evaluated using Mauchly's test where applicable. Effect sizes are reported where applicable (Cohen's *d* for endpoint comparisons; partial *η* ^2^ for repeatedmeasures ANOVA), and exact *p* values are provided in the Results. Although mixed‐effects modelling was considered, repeated‐measures ANOVA was deemed appropriate given balanced group sizes and minimal missingness in longitudinal behavioural data, and because no systematic dropout occurred across time points.

## 3 Results

### 3.1 Characterisation of Clinical–Grade iPSCDerived NPCs

**FIGURE 1:**
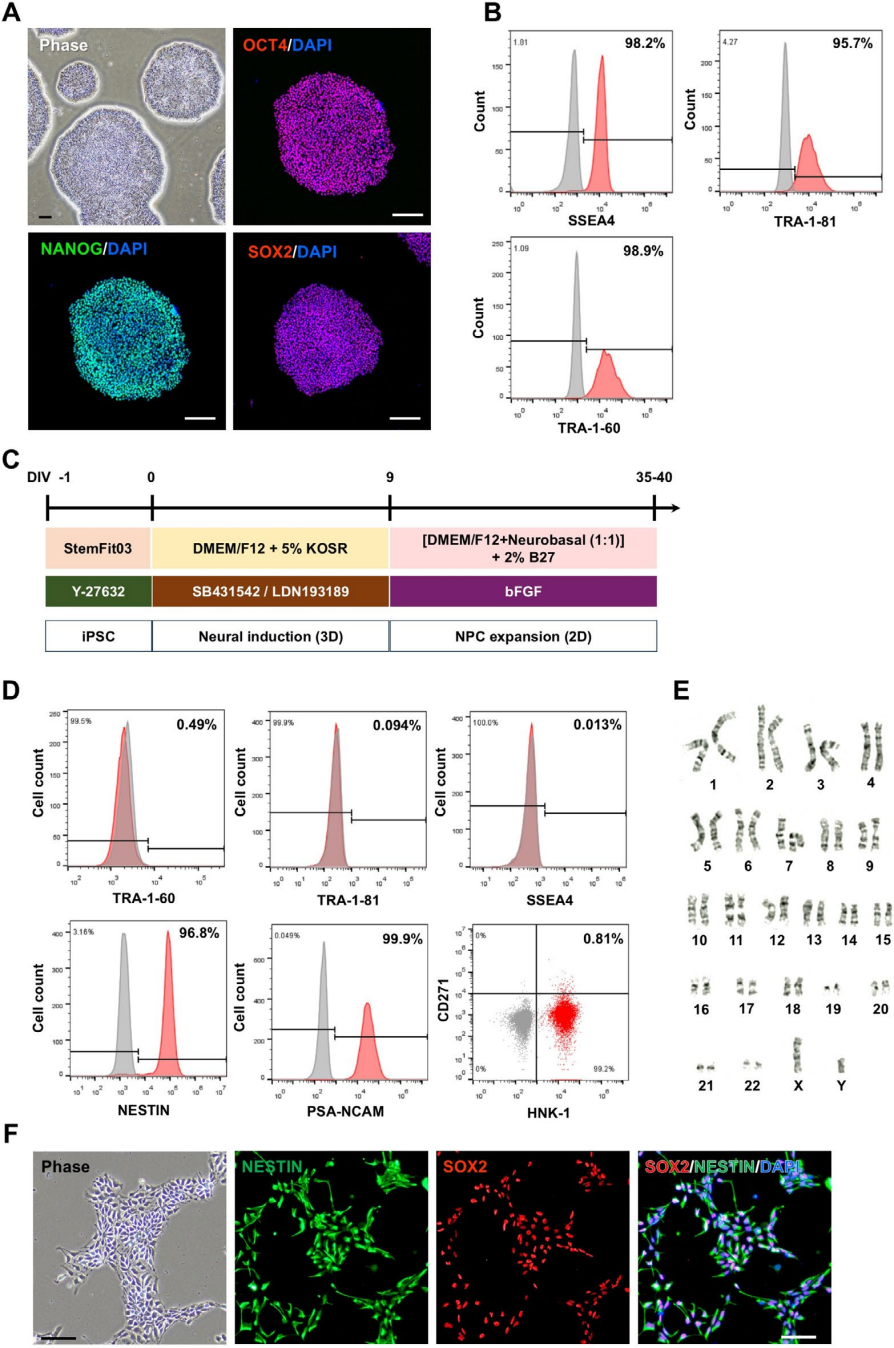
Characterisation of clinical‐grade human iPSC‐derived neural precursor cells (NPCs). (A) Representative bright‐field and immunofluorescence images of undifferentiated YZWJ‐s513 iPSCs showing expression of pluripotency markers OCT4, NANOG, and SOX2 (blue: DAPI). (B) Flow cytometry analysis confirming high expression of pluripotency surface markers SSEA4, TRA‐1‐60 and TRA‐1‐81. (C) Schematic overview of the EB‐based neural induction protocol. (D) Flow cytometry profiles of derived NPCs showing high expression of canonical neural precursor markers NESTIN and PSA‐NCAM, with negligible residual pluripotency marker expression. (E) Normal karyotype of the YZWJ‐s513 line. (F) Immunocytochemistry of NPCs showing robust expression of SOX2 and NESTIN (blue: DAPI). Scale bars: 100 μm.

We first confirmed the identity and pluripotency of the clinical‐grade YZWJ‐s513 iPSC line prior to neural induction. Colonies exhibited typical morphology under phase‐contrast imaging and strongly expressed the core pluripotency markers OCT4, NANOG, and SOX2 (Figure 1A). Flow cytometric analysis demonstrated high expression of the surface antigens SSEA4 (98.2%), TRA‐1‐81 (95.7%), and TRA‐1‐60 (98.9%) (Figure 1B). Neural induction was performed using an EB‐based protocol (Figure 1C), which generated NPCs expressing the canonical NPC markers NESTIN (96.8%) and PSA‐NCAM (99.9%), while showing minimal residual expression of pluripotency‐associated markers (Figure 1D). Karyotypic analysis confirmed chromosomal stability (Figure 1E), and immunocytochemistry further validated SOX2 and NESTIN expression, confirming the NPC identity (Figure 1F).

### 3.2 Transplanted NPCs Survive and Promote Motor Recovery in QA‐Lesioned Rats

**FIGURE 2:**
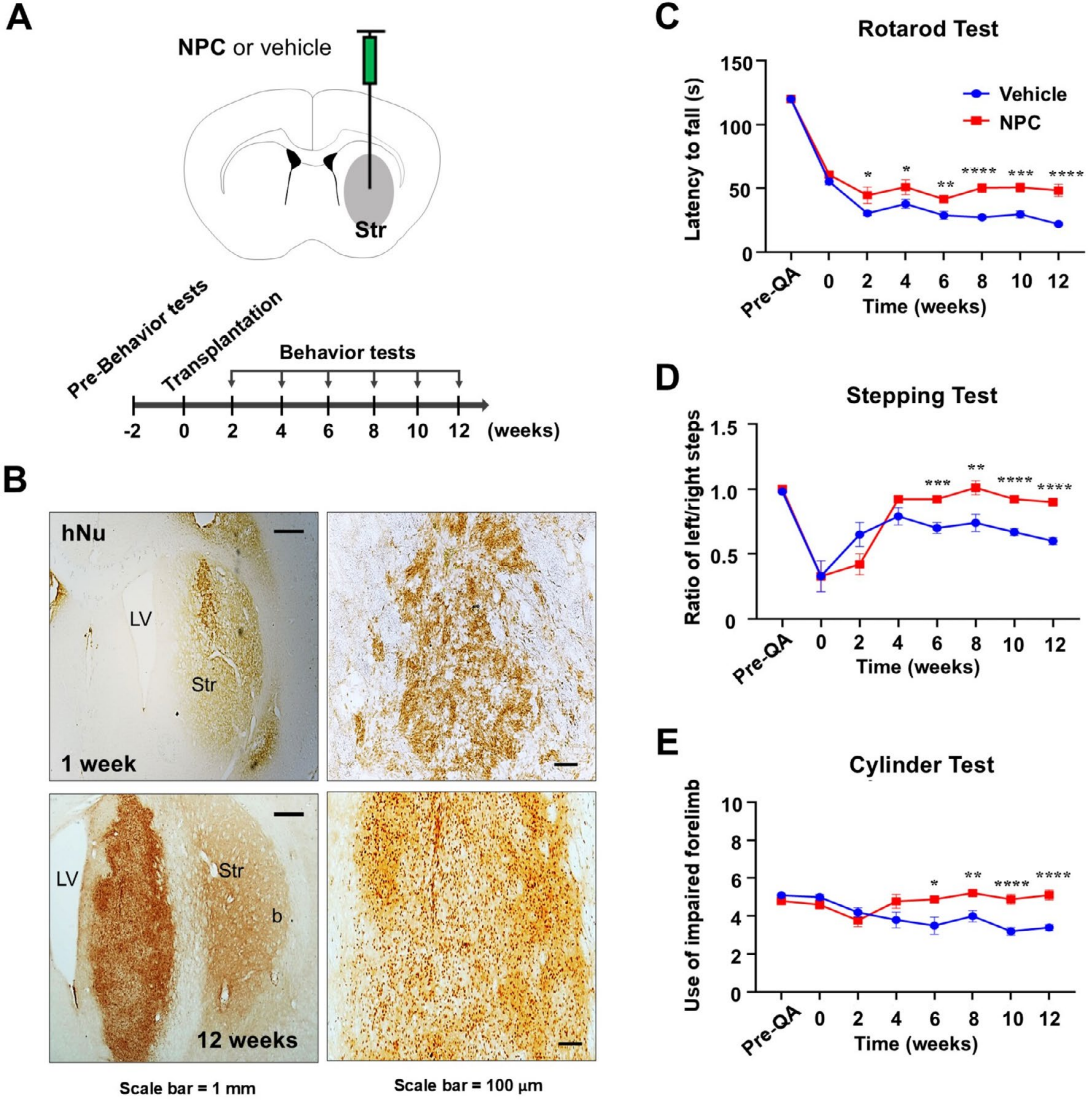
Transplanted NPCs survive and improve motor behaviour in QA‐lesioned rats. (A) Experimental timeline for QA lesion, NPC transplantation, and behavioural testing. (B) Representative images showing grafted human NPCs in the striatum identified by human nuclei (hNuclei) at 1‐ and 12‐weeks post‐transplantation. (C) Rotarod test showing improved latency to fall in NPC‐treated rats. (D) Stepping test demonstrating enhanced forelimb adjusting steps in NPC‐treated rats. (E) Cylinder test showing increased use of the impaired forelimb in the NPC group. Data are mean ± SEM (*n* = 10 per group). *p < 0.05, **p < 0.01, ***p < 0.001, ****p < 0.0001. Statistical significance was determined as described in the Statistical analysis section. Scale bar: 1 mm in low magnification images.

To evaluate therapeutic efficacy, NPCs or vehicles were unilaterally transplanted into the striatum of QA‐lesioned rats. Human‐specific nuclear antigen‐positive (hNuclei^+^) cells were detected at both 1 and 12 weeks post‐transplantation, indicating robust graft survival and distribution within the host striatum (Figure 2B). Motor function was assessed bi‐weekly over 12 weeks using rotarod, stepping, and cylinder tests (Figure 2A). In the rotarod test, the NPC‐transplanted group exhibited significant improvement compared to the vehicle‐injected group from week 2 after transplantation (NPC, *n* = 9; vehicle, *n* = 10; *p* = 0.0408) and maintained this improvement through week 12 (*p* = 0.000047) (Figure 2C). Two‐way repeated‐measures ANOVA revealed a significant treatment × time interaction (*F*(7, 119) = 4.75, *p* = 0.000098, partial *η* ^2^ = 0.218). In the stepping and cylinder tests, motor function in the NPC group began to surpass that of vehicle controls at Week 6 and remained superior through Week 12 (Figure 2D, *p* = 0.00000016; Figure 2E, *p* = 0.000020). In the stepping test, a significant treatment × time interaction was observed (*F*(7, 119) = 5.153, *p* = 0.000049, partial *η* ^2^ = 0.233). In the cylinder test, treatment × time interaction was significant (*F*(7, 119) = 7.374, *p* = 0.00000013, partial *η* ^2^ = 0.303).

### 3.3 In Vivo Differentiation Into Neurons, Astrocytes, and Oligodendrocytes

**FIGURE 3:**
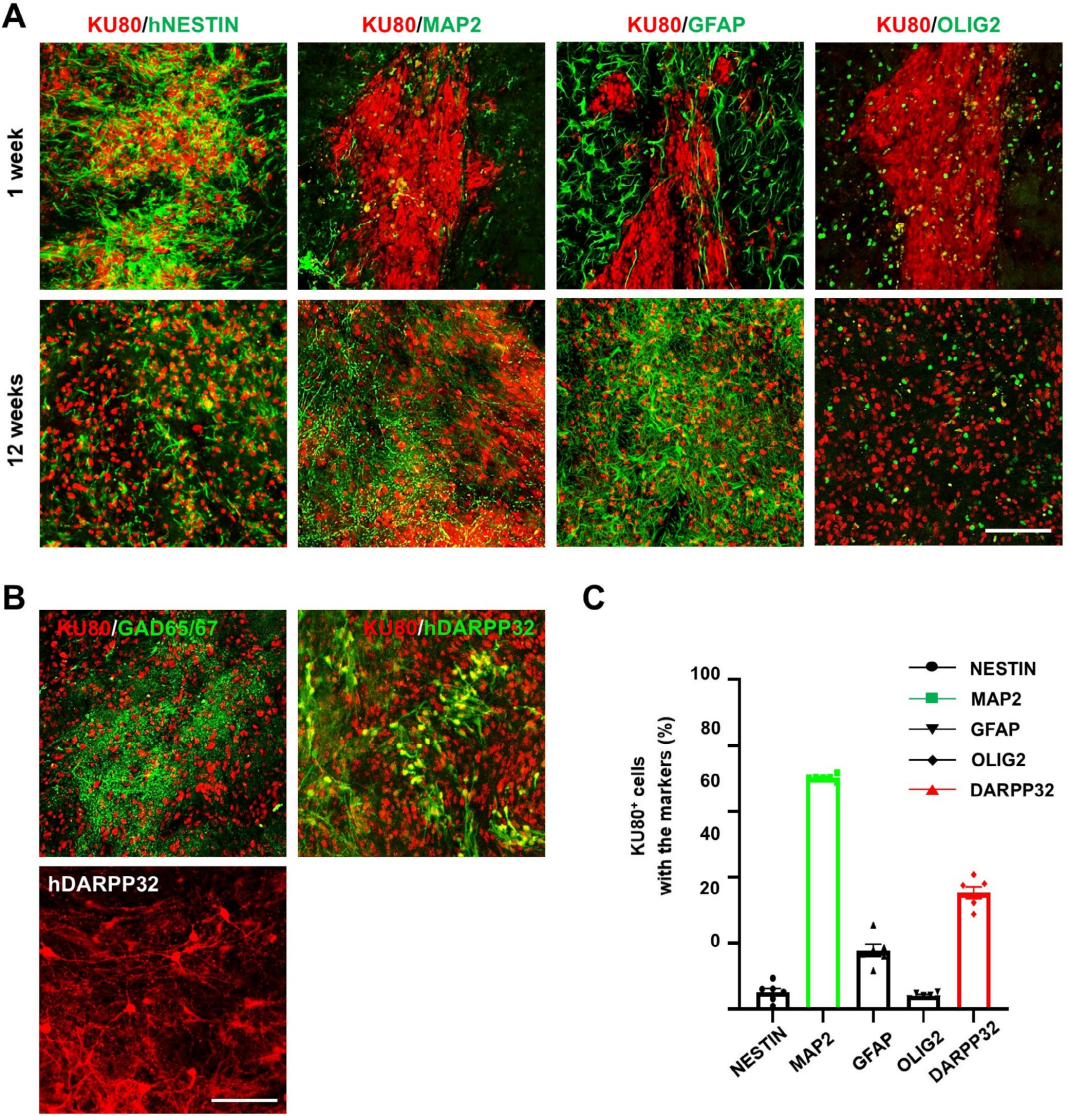
In vivo differentiation of transplanted NPCs into neuronal and glial lineages. (A) Low‐ and high‐magnification images showing KU80^+^ graft‐derived human cells expressing the NPC marker NESTIN at 1 week, and expressing MAP2, GFAP, and OLIG2 at 12 weeks post‐transplantation. Scale bars: Low magnification 1 mm; high magnification 100 μm. (B) Representative images of graft‐derived neurons co‐expressing DARPP32 and GAD65/67 at 12 weeks. (C) Quantification of KU80^+^ graft cells expressing MAP2, GFAP, OLIG2, or DARPP32. Data are mean ± SEM.

We next examined whether transplanted NPCs differentiated into major neural lineages within the host striatum. Double immunostaining for hNuclei and lineage markers showed that at Week 1 post‐transplantation, most donor cells expressed NESTIN, which is an NPC marker (Figure 3A). By Week 12, the majority of donor cells co‐expressed MAP2 (70.19% ± 0.38%), a mature neuronal marker. Subsets of graft‐derived cells also expressed GFAP (17.82% ± 2.07%), an astrocyte marker, and OLIG2 (4.14% ± 0.35%), an oligodendrocyte lineage marker, indicating differentiation into multiple neural cell types (Figure 3A,C). These findings suggest progressive in vivo maturation of the grafted NPCs toward both neuronal and glial phenotypes.

### 3.4 Differentiation Into GABAergic MSNs

To determine whether graft‐derived neurons adopted MSN identity, we assessed the expression of DARPP32, a marker of MSNs, and GAD65/67, a marker of GABAergic neurons, in KU80‐positive donor cells. At 12 weeks post‐transplantation, a substantial proportion of transplanted cells co‐expressed both DARPP32 (35.29% ± 1.93%) and GAD65/67, indicating differentiation into GABAergic MSNs (Figure 3B,C).

### 3.5 Anatomical Integration Into the Striato‐Pallidal Circuit

**FIGURE 4:**
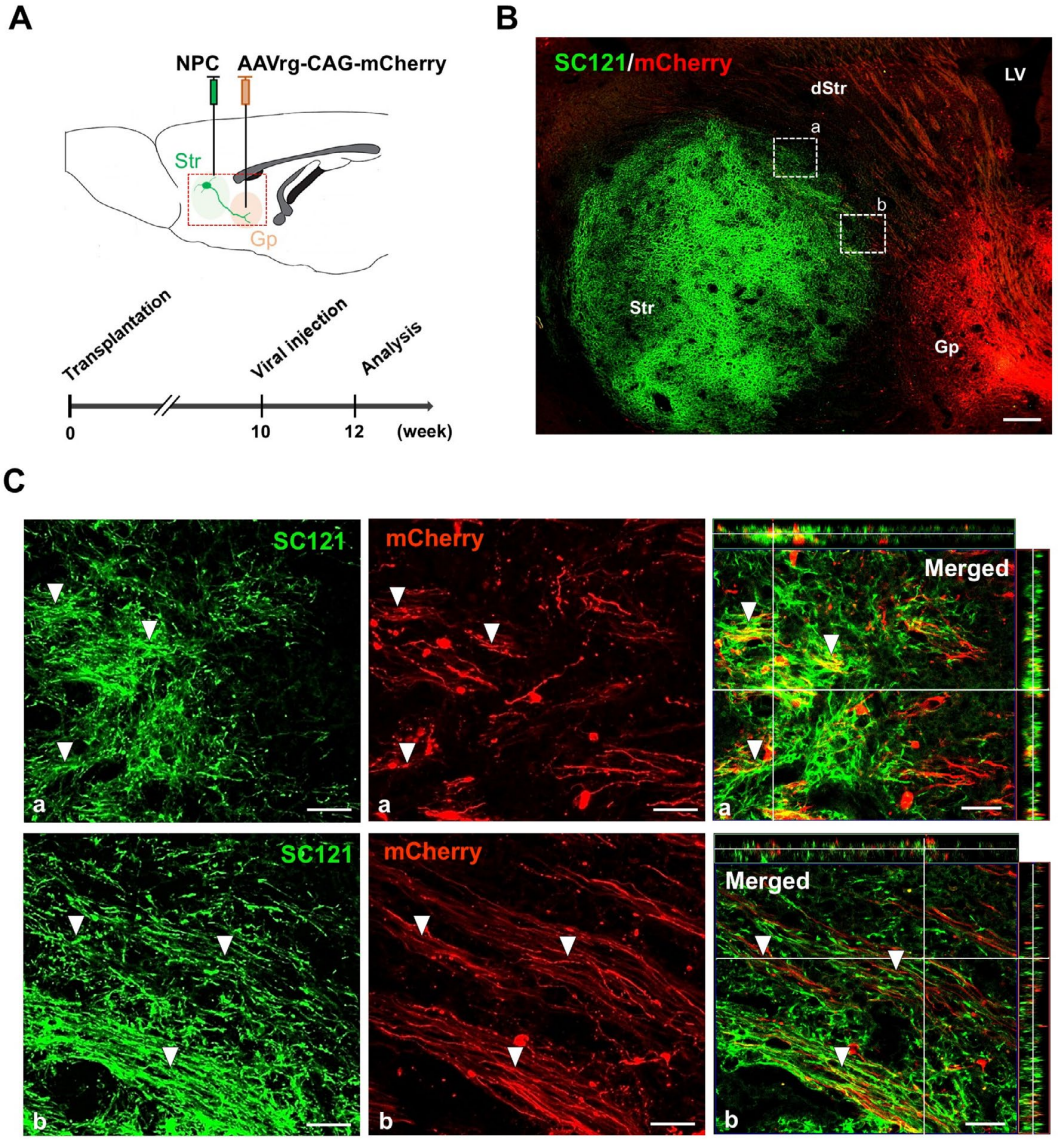
Graft‐derived neurons extend projections to the globus pallidus and structurally integrate into host circuits. (A) Schematic of retrograde AAVrg‐mCherry injection into the globus pallidus (GP). (B) Representative images showing SC121^+^ graft‐‐derived neurons retrogradely labelled with mCherry, indicating axonal projections from graft to GP. (C) High‐magnification images showing SC121^+^ neurites in close apposition to mCherry^+^ host GP neurons, suggesting structural integration. Arrowheads indicate co‐labelled or closely apposed structures. Scale bars: Low magnification 1 mm; high magnification 50–100 μm.

To examine graft‐to‐host connectivity, we employed a retrograde tracing strategy using AAVrg‐CAG‐mCherry injected into the globus pallidus, a major target of striatal GABAergic projections (Figure 4A). Confocal imaging revealed SC121‐positive graft‐derived neurons co‐localized with mCherry, demonstrating that a subset of transplanted neurons extended axons to the globus pallidus and were retrogradely labelled via the host striato‐pallidal pathway (Figure 4B,C). In addition, SC121‐positive neurites were frequently observed in close apposition to mCherry‐positive host neurons, indicating structural incorporation of graft‐derived projections within host circuitry (Figure 4C). In the context of this study, integration refers to axonal targeting and structural incorporation into host projection pathways, rather than direct evidence of synaptic transmission or electrophysiological functional connectivity. Definitive demonstration of functional synaptic integration will require future studies incorporating electrophysiological recordings and trans‐synaptic tracing approaches.

### 3.6 Amelioration of Striatal Atrophy and Ventricular Enlargement

**FIGURE 5:**
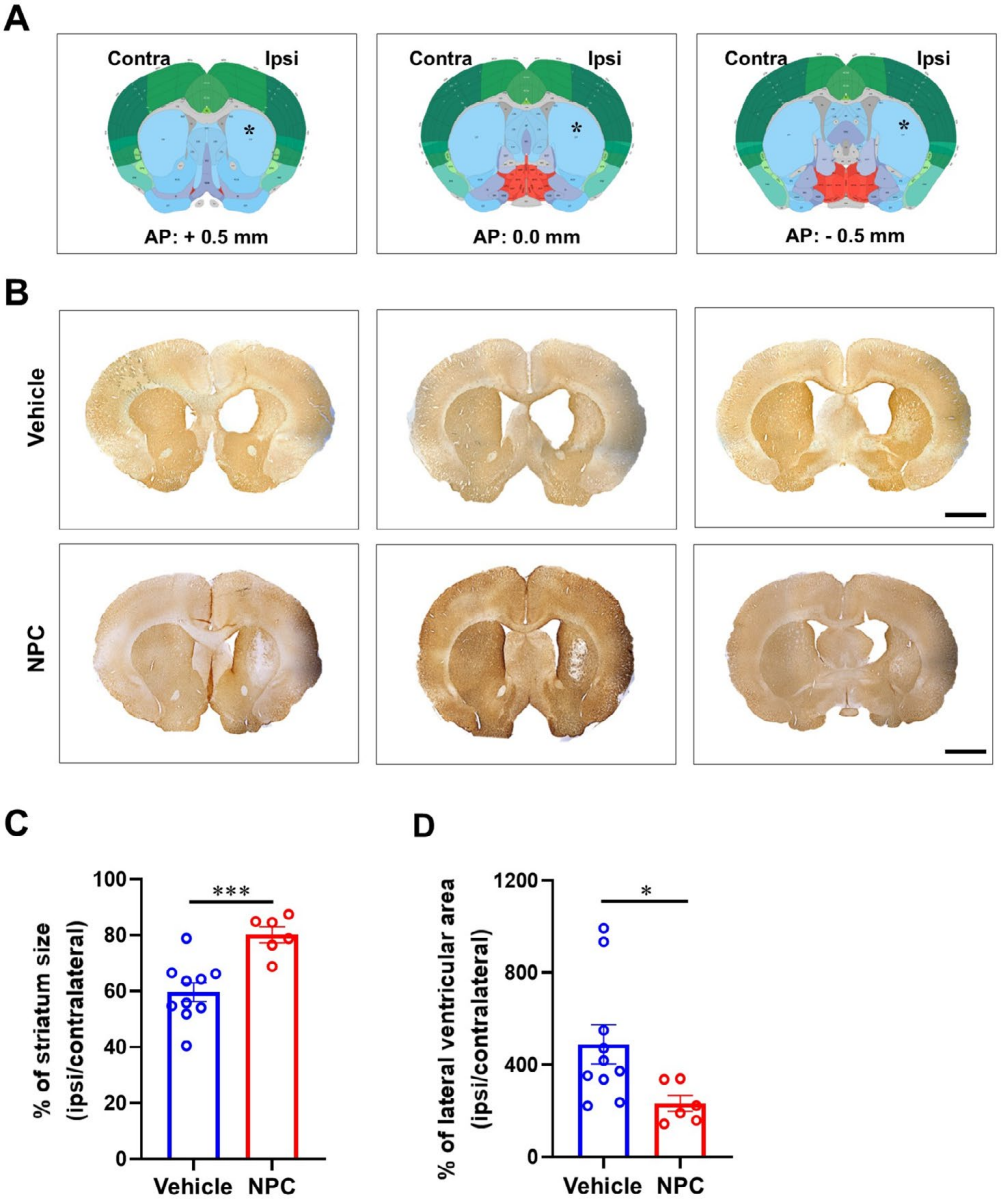
NPC transplantation preserves striatal tissue and reduces ventricular enlargement after QA lesion. (A) Representative coronal sections (AP +0.5 mm relative to bregma) showing DARPP32 immunostaining of striatal tissue in vehicle and NPC‐treated rats. (B–D) Quantification of striatal DARPP32^+^ area ratio (ipsilateral/contralateral) and lateral ventricle size ratio (ipsilateral/contralateral). NPC‐treated rats showed significantly preserved striatal area and reduced ventricular enlargement. Data are mean ± SEM (vehicle *n* = 10; NPC *n* = 6). *p < 0.05, ***p < 0.001. Scale bars: Low magnification 1 mm; high magnification 200 μm.

DARPP32 immunohistochemistry demonstrated marked preservation of striatal architecture in NPC‐transplanted rats compared to vehicle‐injected controls (Figure 5A). Quantitative analysis revealed that the ratio of ipsilateral to contralateral striatal area was significantly higher in the NPC group (Figure 5B,C; NPC, *n* = 6; vehicle, *n* = 10; *p* < 0.001). The ipsilateral‐to‐contralateral striatal area ratio was significantly higher (*t*(14) = 4.23, *p* = 0.0008, Cohen's *d* = 1.13). Ventricular enlargement was significantly reduced, as reflected by a lower ipsilateral‐to‐contralateral ventricular area ratio (Figure 5D; *t*(14) = 2.24, *p* = 0.0416, Cohen's *d* = 0.60).

### 3.7 Reduction of Gliosis and Modulation of Inflammatory Milieu

**FIGURE 6:**
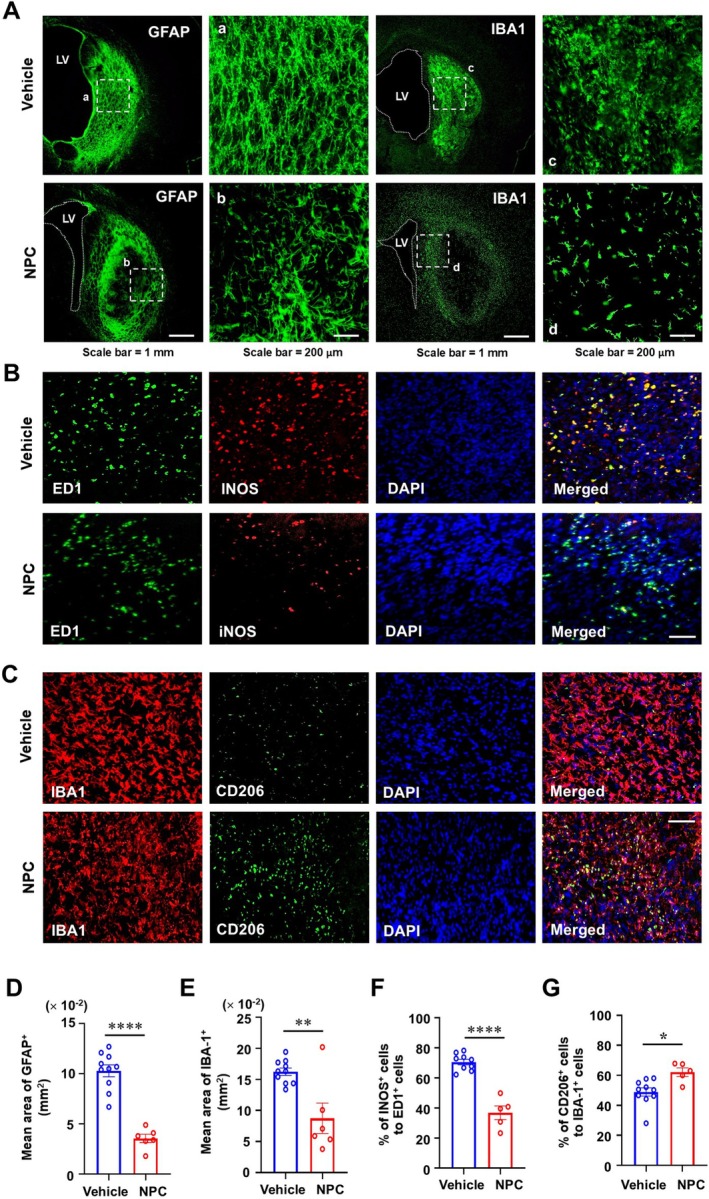
NPC grafts reduce gliosis and shift microglial activation toward an anti‐inflammatory phenotype. (A) Representative GFAP and IBA1 staining showing reduced astrocytic and microglial activation in NPC‐treated striata. (B) Quantification of pro‐inflammatory microglia/macrophages (iNOS^+^/ED1^+^ among IBA‐1^+^ cells) showing a significant reduction in NPC‐treated rats. (C) Quantification of anti‐inflammatory microglia (CD206^+^/IBA1^+^) showing increased proportions after NPC transplantation. (D–G) Summary graphs for gliosis and microglial polarisation indices. Data are mean ± SEM (*n* = 6 NPC; *n* = 10 vehicle). *p < 0.05, **p < 0.01, ***p < 0.001. Scale bars: Low magnification 1 mm; high magnification 200 μm. A: Each lowercase letter corresponds to the respective image in the boxes on the left panel.

Immunostaining for GFAP showed a significant reduction in astrocytic activation within the grafted striatum of NPC‐transplanted rats compared to vehicle controls (Figure 6A,D; *p* < 0.0001). GFAP immunoreactivity was significantly reduced (*t*(14) = 8.06, *p* = 0.00000051, Cohen's *d* = 2.15). IBA‐1 staining similarly revealed reduced microglial activation in the NPC group (Figure 6A,E; *p* = 0.0022). Microglial activation was significantly attenuated (*t*(14) = 3.73, *p* = 0.0022, Cohen's *d* = 1.00). Double labelling for iNOS and ED1 indicated a significant reduction in the proportion of pro‐inflammatory microglia in the NPC‐transplanted striatum (Figure 6B), with quantitative analysis summarised in Figure 6F (*p* = 0.00000003). Conversely, double immunostaining for IBA‐1 and CD206 demonstrated a significant increase in anti‐inflammatory microglia in the NPC group (Figure 6C), as quantified in Figure 6G (*p* = 0.0101), indicating a shift toward a more neuroprotective immune profile. The proportion of iNOS^+^ microglia was significantly decreased (Figure 6F; *t*(14) = 9.46, *p* = 0.000000032, Cohen's *d* = 2.53), whereas CD206^+^ microglia were increased (Figure 6G; *t*(14) = 2.97, *p* = 0.0101, Cohen's *d* = 0.80). Quantitative analyses corresponding to these findings are presented in Figure 6D–G.

## 4 Discussion

In this study, we investigated the therapeutic potential of clinical‐grade, HLA‐homozygous human iPSC‐derived NPCs in a QA‐lesioned rat model of HD. After transplantation into the striatum, the grafted NPCs survived long‐term and differentiated into neurons, astrocytes, and oligodendrocytes. Importantly, a considerable fraction of the neuronal population adopted a DARPP32^+^ GABAergic MSN identity, the cell type most severely affected in HD [5, 34]. Rats receiving NPC grafts showed reduced striatal atrophy, improvements in multiple motor tasks, and a marked attenuation of neuroinflammation. These outcomes suggest that the graft exerts therapeutic effects through a time‐dependent, multifactorial mechanism, in which early behavioural recovery is likely driven predominantly by paracrine neuroprotective and immunomodulatory effects, followed by later contributions from structural incorporation and neuronal replacement as graft‐derived neurons mature within host circuitry. Notably, motor improvement in the rotarod test was observed as early as 2 weeks post‐transplantation, a time point that likely precedes full neuronal maturation and synaptic integration of graft‐derived MSNs. This temporal dissociation supports the interpretation that early functional benefits may arise primarily from modulation of the host microenvironment, including attenuation of excitotoxicity and neuroinflammation, rather than direct circuit reconstruction alone. Consistent with this multifactorial mechanism, we interpret behavioural recovery as potentially reflecting both structural incorporation of graft‐derived neurons into host pathways and paracrine/immunomodulatory effects on the host microenvironment, rather than synaptic replacement alone.

A notable aspect of this work is the use of YZWJ‐s513, a clinical‐grade, HLA‐homozygous iPSC line [35, 36, 37, 38]. This line provides favourable HLA matching for a large proportion of East Asian populations [37, 38], which could reduce the burden of immunosuppression and make allogeneic transplantation more practical [39, 40]. Previous studies have employed human embryonic stem cell (hESC)‐ or iPSC‐derived grafts in HD models [28, 30, 31], but most relied on research‐grade lines, heterogeneous cell populations, or experimental transplantation settings that limit clinical translation. In contrast, our approach utilises GMP‐compliant NPCs derived from a well‐defined, expandable, and genetically stable iPSC line, thereby addressing an important translational barrier and increasing the feasibility of future clinical application. To further support the clinical‐grade positioning, key quality attributes and release criteria for the NPC batch used in vivo are summarised in Table S1.

The transplanted NPCs displayed strong neuronal differentiation capacity: more than 70% of grafted cells expressed MAP2, and a substantial subset acquired DARPP32^+^ MSN characteristics. Retrograde AAV tracing confirmed that graft‐derived neurons extended axons to the host globus pallidus and formed close contacts with mCherry‐labelled host neurons, indicating that grafted neurons engaged with the striatopallidal pathway. This degree of MSN specification and host integration is notable given the variable outcomes reported in earlier transplantation studies. The observation that approximately 35% of grafted cells acquired a DARPP32^+^ MSN phenotype is comparable to or exceeds efficiencies reported in prior studies using fetal tissue or heterogeneous neural progenitor preparations. Importantly, the remaining graft population was composed predominantly of MAP2^+^ neurons and regionally appropriate glial lineages, rather than undifferentiated or non‐neural contaminants. However, these data primarily support structural connectivity (axonal targeting and retrograde labelling) and do not directly demonstrate synaptic or electrophysiological functional integration.

In addition to replacing lost neurons, the grafts also provided neuroprotection. Histological analysis revealed reduced striatal shrinkage and ventricular enlargement, together with diminished astrocytic and microglial activation. Preservation of striatal architecture and mitigation of ventricular enlargement observed in this study may reflect not only local neuroprotection but also compensatory structural plasticity within cortico‐striatal networks, a principle increasingly recognised across diverse neurological and neuropsychiatric conditions. Immunophenotyping further showed a shift toward an anti‐inflammatory profile, characterised by fewer iNOS^+^/ED1^+^ and more CD206^+^/IBA1^+^ microglia/macrophages [41, 42, 43, 44, 45, 46]. These findings are consistent with the known role of astrocytes in restoring glutamate transporters (EAAT1, EAAT2) and Kir4.1 channel function [47, 48, 49, 50, 51, 52], processes that help prevent excitotoxicity. Our earlier work in the YAC128 HD mouse model also demonstrated that iPSC‐derived astrocytes can normalise Kir4.1 expression and glutamine synthetase activity [53], supporting the contribution of the astrocytic component to the observed protective effects.

Compared with recent studies using human neural stem cells or fetal progenitors [28, 30, 32, 33], the defined NPCs used here combine reliable MSN generation, demonstrated circuit connectivity, and immunomodulatory activity. The availability of a clinical‐grade, HLA‐homozygous line further addresses ethical concerns and scalability issues that have hindered fetal tissue–based approaches, offering a reproducible platform for clinical development.

This study has several limitations. First, because xenotransplantation into an immunologically mismatched rat host necessitated continuous CsA administration, the immunomodulatory effects of systemic immunosuppression must be considered when interpreting glial phenotypes. Importantly, both NPC‐transplanted and vehicle‐treated animals received identical CsA regimens, indicating that the observed differences in microglial polarisation reflect graft‐associated effects superimposed on a shared immunosuppressive background. In future clinical settings involving HLA‐matched or HLA‐compatible allogeneic transplantation, the intensity and duration of systemic immunosuppression may be substantially reduced or transient, potentially allowing graft‐derived immunomodulatory effects to manifest more distinctly. Second, the observation period was limited to 12 weeks, so longer‐term safety, including tumorigenicity and durability of functional benefit, was not assessed. Third, our behavioural assessments focused on motor deficits in a unilateral lesion model and did not address the full spectrum of cognitive and psychiatric features seen in patients with HD. Because the QA‐lesioned model and behavioural battery employed here primarily assess motor dysfunction, the present study does not address cognitive or psychiatric symptoms that critically impact patient quality of life in HD, which should be explored in future models and clinical studies. Future studies in humanised and large‐animal models with extended follow‐up will be essential to address these issues. Moreover, continuous CsA administration may have influenced host neuroinflammatory readouts; therefore, immune dynamics in HLA‐matched or transiently immunosuppressed settings may differ and should be addressed in future translational studies. No evidence of aberrant graft overgrowth, proliferative clusters, or tumour‐like structures was observed within the 12‐week observation period; however, this duration is insufficient to fully exclude late‐onset proliferative risks associated with iPSC‐derived products. Accordingly, longer‐term safety assessments in extended‐duration studies and large‐animal models will be essential to rigorously evaluate tumorigenicity and durability prior to clinical translation. In addition, the QA‐lesion model recapitulates key aspects of striatal degeneration and motor dysfunction but does not capture the progressive genetic and multisystem pathology of HD; thus, validation in genetic and humanised models will be important to strengthen translational relevance. Finally, while graft survival, differentiation, and behavioural recovery were observed concurrently, the present study was not powered to establish definitive predictive relationships between individual graft parameters and functional outcomes; exploratory graft–behaviour correlation analyses are provided in Figure S1 and should be interpreted cautiously.

In conclusion, our findings provide preclinical evidence that transplantation of clinical‐grade, HLA‐homozygous iPSC‐derived NPCs can achieve long‐term survival, multilineage differentiation, MSN replacement, host circuit integration, and modulation of neuroinflammation in HD. Future work should focus on long‐term efficacy and safety studies in HLA‐matched humanised mice and non‐human primates to establish the foundation for clinical translation in HD patients.

## Author Contributions

H. Jeon, I.‐S. Lee and J. Song designed the study. H. Jeon and S. Lee performed animal and histopathological experiments. H.C. Park and B. Kim prepared YZWJ s513‐NPCs. H. Jeon and I.‐S. Lee performed statistical analyses. H. Jeon and I.‐S. Lee wrote the manuscript. H. Jeon, H.S. Kim, I.‐S. Lee, and J. Song interpreted and discussed the results. J. Song supervised the entire study, provided financial support, critically revised the manuscript, and approved the final manuscript.

## Funding

This research was supported by the Korean Fund for Regenerative Medicine (KFRM), funded by the Ministry of Science and ICT and the Ministry of Health & Welfare (RS‐2022‐00070674, RS‐2024‐00333096); the Korea Institute for Advancement of Technology (KIAT) grant funded by the Ministry of SMEs and Startups (RS‐2024‐00488530); the Korea Health Technology R&D Project through the Korea Health Industry Development Institute (KHIDI), funded by the Ministry of Health & Welfare (RS‐2022‐KH129581) and internal funding from iPS Bio Inc.

## Conflicts of Interest

H.C. Park, B. Kim and I.‐S. Lee are employees of iPS Bio Inc. J. Song is the founder of iPS Bio Inc. The other authors declare no conflicts of interest.

## Supporting information


**Figure S1:** Exploratory correlation analysis between graft survival and motor performance outcomes. Scatter plot illustrating the association between graft survival, quantified as the total hNuclei^+^ graft area (mm^2^) per animal, and endpoint motor performance assessed by the latency to fall in the rotarod test. Each data point represents an individual transplanted rat. Pearson's correlation analysis revealed a significant positive correlation between graft area and motor performance (*r* = 0.82, *p* = 0.046). Given the limited sample size, this correlation analysis is exploratory and not powered to establish definitive predictive or causal relationships.

**Table S1:** Key quality attributes and release criteria for the clinical‐grade iPSC‐derived NPC batch used for in vivo transplantation. All release testing was performed according to GMP‐compliant standard operating procedures prior to transplantation. The values shown correspond to the specific NPC batch used in the present study.

## Data Availability

The data that support the findings of this study are available from the corresponding author upon reasonable request.
