# Supplementary figures and images for: Clinical‐Grade Human Induced Pluripotent Stem Cell‐Derived Neural Precursor Cells Restore Motor Function and Preserve Striatal Integrity in a Quinolinic Acid‐Lesioned Rat Model of Huntington's Disease
Source: Cell Prolif. 2026 Feb 26;59(7):e70189. doi: 10.1111/cpr.70189 (PMC13325475; doi:10.1111/cpr.70189)

## Rotarod test

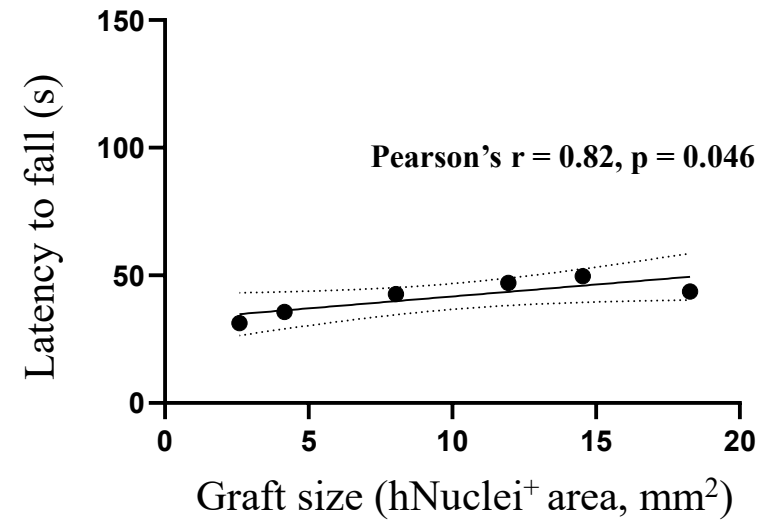

Supplement: Supplementary file 1 — Figure S1: Exploratory correlation analysis between graft survival and motor performance outcomes. Scatter plot illustrating the association between graft survival, quantified as the total hNuclei+ graft area (mm2) per animal, and endpoint motor performance assessed by the latency to fall in the rotarod test. Each data point represents an individual transplanted rat. Pearson's correlation analysis revealed a significant positive correlation between graft area and motor performance (r = 0.82, p = 0.046). Given the limited sample size, this correlation analysis is exploratory and not powered to establish definitive predictive or causal relationships. [file CPR-59-e70189-s001.pdf]
