# Supplementary material for: Clinical‐Grade Human Induced Pluripotent Stem Cell‐Derived Neural Precursor Cells Restore Motor Function and Preserve Striatal Integrity in a Quinolinic Acid‐Lesioned Rat Model of Huntington's Disease
Source: Cell Prolif. 2026 Feb 26;59(7):e70189. doi: 10.1111/cpr.70189 (PMC13325475; doi:10.1111/cpr.70189)
Supplement: Supplementary file 2 — Table S1: Key quality attributes and release criteria for the clinical‐grade iPSC‐derived NPC batch used for in vivo transplantation. All release testing was performed according to GMP‐compliant standard operating procedures prior to transplantation. The values shown correspond to the specific NPC batch used in the present study. [file CPR-59-e70189-s002.docx]

| **Parameter** | **Method** | **Acceptance criteria** |
| --- | --- | --- |
| Viability | Trypan blue | ≥ 80% |
| Endotoxin | LAL assay | < 0.5 EU/mL |
| Sterility | BacT/ALERT | Pass |
| Mycoplasma | qPCR | Negative |
| Residual pluripotency (OCT4/TRA-1-60) | Flow cytometry | < 0.1% |
| Identity (NESTIN/PSA-NCAM) | Flow cytometry | > 90% |
| Karyotype | G-banding | Normal |

**Supplementary Table S1.** Key quality attributes and release criteria for the clinical-grade iPSC-derived NPC batch used for *in vivo* transplantation.
